# Using mobile phones and social media to facilitate education and support for rural-based midwives in South Africa

**DOI:** 10.4102/curationis.v38i2.1500

**Published:** 2015-12-14

**Authors:** Jennifer Chipps, Christoph Pimmer, Petra Brysiewicz, Fiona Walters, Sebastian Linxen, Thandiwe Ndebele, Urs Gröhbiel

**Affiliations:** 1 School of Nursing, University of the Western Cape, South Africa; 2 Sydney Nursing School, University of Sydney, Australia; 3 Institute for Information Systems, HSW, University of Applied Sciences and Arts North-Western Switzerland, Switzerland; 4 School of Nursing and Public Health, College of Health Sciences, University of KwaZulu-Natal, South Africa

## Abstract

**Background:**

Empirical studies show the value of mobile phones as effective educational tools to support learning in the nursing profession, predominantly in high income countries.

**Problem statement:**

The rapidly increasing prevalence of mobile phone technology in Africa nourishes hopes that these tools could be equally effective in lowly resourced contexts, specifically in efforts to achieve the health-related Millennium Development goals. The purpose of this study was to investigate the perception and use of mobile phones as educational and professional tools by nurses in lowly resourced settings.

**Methodology:**

A quantitative survey using self-administered questionnaires was conducted of rural advanced midwives.

**Results:**

Fifty-six nurses (49.6%) from the 113 rural-based midwives attending an advanced midwifery training programme at the University of KwaZulu-Natal, South Africa, filled in a questionnaire. The results showed that, whilst nurses regarded their technology competences as low and although they received very little official support from their educational and professional institutions, the majority frequently used mobile functions and applications to support their work and learning processes. They perceived mobile devices with their voice, text, and email functions as important tools for the educational and professional activities of searching for information and engaging with facilitators and peers from work and study contexts. To a lesser extent, the use of social networks, such as WhatsApp and Facebook, were also reported.

**Conclusion and recommendation:**

It is concluded that educational institutions should support the appropriate use of mobile phones more systematically; particularly in relation to the development of mobile network literacy skills.

## Introduction

As part of a strategy to address three of the Millennium Development goals to improve child and maternal health and fighting HIV, rural-based registered midwives have been enrolled in a part-time, two-year, Advanced Midwifery Programme at the University of KwaZulu-Natal, South Africa.

## Background and selective literature review

### Challenges of health workers in rural areas and the potential of mobile technology

In rural and disadvantaged areas in lower- and middle-income countries, health workers have limited access to education, up-to-date knowledge, and professional networks. These conditions contribute, amongst others, to professional isolation, attrition, poor performance, and emigration (HIFA Report 2010, Hongoro & McPake 2004, WHO 2010). In these settings the mobile phone is often the most reliable technology for health care providers to perform their work (Crow *et al.* 2012). In fact, in low- and middle-income countries, the use of mobile phones has dramatically increased and is considered to have a large impact on developmental issues. In South Africa this increase includes 94.2 mobile cellular subscriptions per 100 people (The World Bank 2011) and a mobile broadband penetration that had reached 29% by the end of 2013 (ITU 2014).

In this light, United Nations organisations, such as UNICEF, ITU and the World Bank, have great expectations for the potential of mobile phones to support and educate health care providers (Holmes 2010, ITU 2010, The World Bank 2012), and educational institutions have started piloting these tools (Chang *et al.* 2011).

## Mobile learning and communication

Mobile phones, similar to the old digital assistants, form new kinds of communication and interaction that can also define novel approaches to teaching and learning (De Marcos Ortega *et al.* 2011, McLeod & Mays 2008). In nursing education and midwifery, smartphones can be used for quick access to educational materials and guidelines during clinical and class activities or clinical conferences, a range of applications expand the smartphone functions even further (Havelka 2011, Phillippi & Wyatt 2011). In a number of studies, mobile phones are reported to be used for educational purposes in clinical contexts to access learning materials (Clay 2011) or social support at the bedside (Young *et al.* 2010). However, some barriers to the use of mobile phones in nursing have been recorded, including cost, disease transmission, equipment interference (Phillippi & Wyatt 2011), and ethical issues of data security.

However, despite the increasing pentetration of mobile devices in low- and middle-income countries, mobile learning research seems to focus on learning at schools, universities, workplaces, or on life-long learning in high income areas (Hwang & Tsai 2011; Wu *et al.* 2012). There is still little solid knowledge available about how to use mobile media to effectively support health workers in disadvantaged areas in low- and middle-income countries (Braun *et al.* 2013; Deglise, Suggs & Odermatt 2012; Mechael *et al.* 2010; Tomlinson *et al.* 2013). In these settings, Traxler and Kukulska-Hulme (2005) consider the potential of mobile learning to provide education without dependence on extensive traditional communications infrastructure. However, it is argued that current perceptions about mobile learning and development seems to be oversimplified and techno-centred (Traxler 2012; Winters 2013). Similarly, most health studies focus on small-scale pilot interventions, adopt a ‘techno-optimistic’ view, do not pay attention to socio-cultural practices and have a limited theoretical foundation. Beyond interventions, published literature on the gradual growth and appropriation of mobile phones for health and educational purposes is scarce (Chib 2013; Chib, Van Velthoven & Car 2014).

Midwifery studies in Indonesia point to the potential of cell phones. Their use is positively associated with access to institutional resources (Lee, Chib & Kim 2011), their capacity to solve patient-related challenges, the enhancement of peer interaction (Chib & Chen 2011), the empowerment and respect that midwives enjoy within their communities (Chib & Chen 2011), and higher levels of health knowledge (Lee *et al.* 2011). These positive outcomes have been realised in a non-linear approach resulting from the dialectical tensions amongst the interactions of benefits and constraints, such as gender or midwifery roles, a lack of technical competency and resource control in traditional social hierarchies (Chib & Chen 2011).

### The use of mobile technology to access social media and social networks

Internet-enabled mobile phones increasingly permit users to access social network sites. Indeed, statistics allow the conclusion that smart mobile phone users spend considerably more time on social media and social network sites than users of personal computers (Favell 2010). Again, medical and health research has concentrated on the educational use of social media in high income countries (Gray, Annabell & Kennedy 2010). Their findings identify educational benefits as well as risks regarding ethical issues and privacy. However, very little research has been conducted about the use of social software on mobile phones in developing or transitional countries, though their potential is considered to be high (Kolko, Rose & Johnson 2007). Only a few studies have analysed the rich educational potential of the convergence of mobile phones and social networking sites for nursing and medical education in developing countries. A study, for example, illustrates how Asian health professionals are using social network sites on their mobile phones to deliberately engage with explicit forms of educational content, such as quizzes and case presentations, as well as to participate in virtual professional communities that allow for the announcement and negotiation of occupational status and professional identities. Such technologies permit the users’ educational engagement beyond local communities and facilitate loose and ephemeral connections to professional (health) networks (Pimmer, Linxen & Gröhbiel 2012a; Pimmer *et al.* 2013). A qualitative study in a similar programme setting as this investigation concluded that midwives are using their mobile phones to support authentic problem solving in relation to critical patient cases to facilitate reflective practice and to establish social presence in the form of emotional support and inclusion (Pimmer *et al.* 2014). As a result of the prevailing qualitative findings it can be concluded that future research must concentrate more attention on analysing such applications. Further research is required to address the extent to which the engagement with such technologies impacts learning and communicating health information.

### Problem statement

More than 100 midwives enrolled in a two-year Advanced Midwifery Programme at five rural sites in the KwaZulu-Natal Province of South Africa. The challenge for the teaching staff of the programme was to connect these midwives to the university in real time and to ensure that they had ready access to all learning materials and resources. Though lectures were provided by means of videoconferencing and midwives reported that they had access to computers, it was clear that uptake of the learning management systems had been low (Chipps *et al.* 2015). A previous study concluded that, despite the high possibility of phone ownership (Pimmer *et al.* 2014), it was not clear what the use for, and perceptions about, the functionality of mobile phones for educational and work-related activities had been.

### Definition of key concepts

**ICTs:** ICTs are information and communication technologies that include hardware, such as computers (desktop and laptop) and mobile phones (smartphones and traditional mobile phones), as well as software applications and communication functions, such as calling and messaging.

**Mobile phones:** A subset of ICTs includes smartphones (e.g. iPhone, Blackberry) that have additional functions to basic phoning and SMS (text messaging), such as Internet access and a range of applications, and mobile phones without Internet access.

**Facilitators:** Facilitators were Registered Educators and Midwives employed by the university to support students clinically and academically in rural areas.

## Research objectives of the study

The research objectives were to establish existing usage patterns and perceptions of ICT and, in particular, of mobile phones and social media and networks, e.g. Facebook to gain a better understanding for the purpose of incorporating these technologies into existing educational programmes.

## Research method and design

### Context of the study

The context of the study was the Advanced Midwifery Training Programme at the University of KwaZulu-Natal, South Africa, which was presented at five remote rural hospital sites and one university site. Because the midwifery students were living, working, and studying locally a blended educational programme had been designed to address their educational needs with the aim of not removing them from their local setting for educational purposes. That included weekly in-person videoconferencing lectures from the university to the local rural hospitals and the appointment of local facilitators to support the midwives at those rural sites. Programme material in four subjects had also been placed online by utilising an open source learning management system; however, most of the material and resources were photocopied and posted to the rural sites.

### Design

A quantitative survey on the use and effectiveness of mobile phones, computers and social networks for work and educational purposes was conducted in the second half of 2012 by means of a structured questionnaire. This survey was the second component of a mixed method study to investigate mobile phone and social media usage for educational purposes in lowly resourced settings (Pimmer *et al.* 2014).

### Sample and population

The population of the study comprised 113 advanced midwifery nursing students enrolled in the two-year programme at the time of the survey; 69 respondents were second year students whilst 44 of them were first year students. No sampling was done and all midwives were eligible to participate in the study, resulting in 56 valid respondents (49.6% response rate): 45 from the second year (65.2%) and 11 from the first year (25.0%). Participation varied across sites, ranging from 100% at one rural site to 27.5% at the university attendance site (Table 1).

**TABLE 1:** Respondents of the study.

Site	Couse respondents	Sample (%)
Site A	13	13 (100)
Site B	17	13 (76.5)
Site C	12	5 (41.7)
Site D	20	11 (55.0)
University site	51	14 (27.5)
**Total**	**113**	**56 (49.6)**

### Materials

The questionnaire was developed and refined based on the findings of previous qualitative studies, mainly from an interview-based investigation from the same programme setting (Pimmer *et al.* 2014) and, additionally, from a qualitative study from a different low resourced context (Pimmer, Linxen & Gröhbiel 2012b). In addition, items were adopted from an extensive survey with established validity and reliability that had examined the use and perception of information technology by undergraduate students (EDUCAUSE 2011). The questionnaire was in English and took approximately 20 to 30 minutes to complete.

### Data collection and analysis methods

The questionnaires were distributed by the local facilitators and then returned to the research staff at the university. Data were entered in the SPSS version 22.0 computer program and analysed using appropriate descriptive and non-parametric statistics. Frequency of mobile phone usage was estimated out of a possible 365 days a year, with responses assigned the following estimated values to convert frequency of use to a numeric variable: ‘using it more than once a day’ = 356, ‘more than once a week’ = 208 (52 weeks x 4 times a week), ‘more than once a month’ = 48 (12 months x 4 times a month), and ‘once a month’ = 12. Four times was taken as an estimate of use per week or month. Inapplicable data were excluded and missing data were included.

### Ethics

The study received ethical clearance from the Human and Social Science Ethics Committee from the University of KwaZulu-Natal and permission was granted by the School of Nursing at the university to conduct the study. Written informed consent was obtained, anonymity of respondents was maintained and participation in the study was voluntary. Data were not able to be traced back to the respondent.

## Results

### Description of sample

Fifty-six midwives (49.6%) from four rural sites and one site attached to a university responded to the survey (Table 1); 42 from the second year of training (60.9%) and 11 from the first year of training (25.0%). Of the 56 respondents, only two midwives reported that they were male. The average age was 42.5 years (SD 75) with a range between 30 and 57 years. Only six respondents (10.7%) reported having completed a degree in nursing with the rest having completed diplomas in nursing.

### General mobile phone use

Though respondents in this study did report having access to computers (*n* = 48; 85.7%), nearly a quarter of them (*n* = 13; 23.2%) had no computer access at home. In contrast, all respondents excepting two (96.4%) reported owning a mobile phone. Twenty-eight (50.0%) of the respondents reported owning a smartphone. The mobile phone was used most frequently for making phone calls with an estimated average use of 302 days a year (SD 108.9); 41 respondents (73.2%) reported that they made phone calls several times a day. That was followed by SMSs or texting with an estimated use of 250 days a year (SD 135.8). Twenty-nine (51.8%) respondents reported that they sent text messages several times a day; respondents reported sending an average of 4.7 (SD 5.2, median 3, range 0–27) outgoing messages per day and receiving an average of 6.2 (SD 6.4, median 5, range 0–30) messages per day. The WhatsApp messaging application had a general estimated use of 181.8 days a year (167.4) and was used several times a day by 13 (23.2%) respondents; Facebook was used for 127.9.8 days a year (143.8) and was used several times a day by 6 respondents (10.7%); email had an overall general estimated use of 101.6 days a year (143.5) and was used several times a day by 7 respondents (12.5%). There were significant differences by age group for how often they used Facebook functionalities, with only one of the 9 respondents over 50 years old reporting using Facebook at all and then only about once per month (*X *^2^ = 20.1 [df = 8],* p* = .002).

### Current use of mobile phones and social networks to support learning

**Mobile phones**: When examining how mobile phones were used to support learning, three areas were identified: (1) contacting facilitators or peers to discuss topics and tasks from the course, (2) contacting facilitators or peers to discuss work-related topics and tasks, and (3) searching for information and using mobile phones in the clinical context.

Forty-five (80.4%) respondents thought mobile phones were important for contacting programme facilitators, 38 (67.9%) for contacting colleagues, and 40 (71.4%) for contacting their peers or fellow students. The majority of the respondents (*n* = 47; 83.9%) thought they were important for searching for work-related information, whilst 40 (71.4%) respondents reported using them at least several times a week. Though 31 (55.4%) thought mobile phones were important for work-related clinical care, mobile phones were seldom used to document or share cases by means of images (*n* = 11; 19.6%).

The most frequent activity reported by respondents was contacting fellow students by phone call (*n* = 31; 55.4%) followed by SMS messages (*n* = 26; 46.4%) to discuss study issues. Nearly half (*n* = 25; 44.6%) used phone calls to contact facilitators for both study and work-related reasons (Table 2).

**TABLE 2:** Mobile phone functionalities used (*N* = 56).

Variable	Programme related		Work-related		
	Facilitators (%)	Students (%)	Facilitators (%)	Colleagues (%)	Students (%)
Phone calls	25 (44.6)	31 (55.4)	25 (44.6)	22 (39.3)	24 (42.9)
SMSs	15 (26.8)	26 (46.4)	14 (25.0)	22 (39.3)	17 (30.4)
Emails	9 (16.1)	6 (10.7)	8 (14.3)	8 (14.3)	10 (17.9)
Facebook	3 (5.4)	3 (5.4)	3 (5.4)	4 (7.1)	4 (7.1)
WhatsApp	6 (10.7)	9 (16.1)	7 (12.5)	8 (14.3)	6 (10.7)

**Social networks: Facebook:** The use of Facebook as a social network application on either mobile phone or computer was specifically evaluated as a potential new application to use for educational activities. Regarding the current use of Facebook at the time of the survey, 17 respondents (30.4%) reported accessing social network sites, such as Facebook, using their laptops and 20 respondents (35.7%) reported accessing social networks using their smartphones. Twenty-three respondents (41.1%) agreed that social networking sites were becoming important. However, the use of social networks to reflect on work-related experiences was low with only 10 (17.9%) respondents who reported that they were using social networking sites for that purpose. Obtaining information for medical and clinical support was even lower with only small numbers of respondents using Facebook at least several times a week: for example reading messages from Facebook friends on medical and clinical topics (*n* = 7; 12.5%), sharing or accessing medical and clinical resource lines in chats (*n* = 6; 10.7%), as well as sharing medical experiences in chats (*n* = 5; 8.9%), comments (*n* = 4; 7.1%), participation in quizzes (*n* = 2; 3.6%), clinical case presentations (*n* = 2; 3.6%), and posts (*n* = 2; 3.6%).

### Possibilities for mobile phone and social networking use in the education of midwives in rural communities

The respondents’ perceptions of the use of technology were examined with regard to the potential of using mobile phones and social networking activities such as Facebook to provide access to information and support learning. The characteristics of the institution (educational learning environment and ICT support) were also examined.

## Institutional learning environment characteristics

**Teaching related activities:** In respect of the potential to enhance the current learning environment using mobile technology, the most prevalent learning activities in respondents’ educational programme were teamwork and problem solving (*n* = 45, 80.4%), which were identified as activities that could be enhanced through access and use of ICTs (Table 3).

**TABLE 3:** Institutional environment (*N* = 56).

Variable	Agreement (%)
**Teaching activities**	
Lecturers include teamwork	45 (80.4)
Independent problem solving is encouraged	45 (80.4)
Lecturers engage students in discussions	41 (73.2)
Lectures consist of formal presentations	36 (64.3)
Lecturers provide feedback about individual performance	36 (64.3)
**Facilitators used the following ICTs**	
Photo camera	22 (39.3)
Desktop computer	24 (42.9)
Mobile phone	31 (55.4)
Video camera	32 (57.1)
Smartphone	39 (69.6)
E-learning sites & course websites	39 (69.6)
Learning Management Systems	47 (83.9)
Laptop	47 (83.9)
**ICT support at the institution for mobile phones and social network sites**	
The university supports the use of mobile phones for learning	34 (60.7)
The university supports the use of social networking sites for learning	22 (39.3)
The use of social network sites for educational purposes is being taught as part of nursing education	17 (30.4)
The use of mobile devices for educational use is being taught as part of nursing education	14 (25.0)
**ICT support at work for mobile phones and social networking sites**	
The use of mobile devices for educational use is supported at work	14 (25.0)
The use of social networking sites for educational use is supported at work	9 (16.1)

Respondents were asked to identify which ICT facilitators should be used more frequently, with the highest support indicated being for texting (*n* = 32; 57.1%), followed by phone calls (*n* = 30; 53.6%), MS PowerPoint presentations (*n* = 27; 48.2%), learning management systems (*n* = 23; 41.1%), e-learning programmes (*n* = 18; 32.1%), WhatsApp and emailing (*n* = 14; 25.0 %), MS Word documents (*n* = 13; 23.2%), eBooks (*n* = 13; 23.2%), video sharing sites (*n* = 12; 21.4%), Skype (*n* = 11; 19.6%), bookmarking on an Internet browser (*n* = 10; 17.9%), Internet chats (*n* = 9; 16.1%), wikis and virtual worlds (*n* = 6; 10.7%), Facebook (*n* = 5; 8.9%), podcasts (*n* = 4; 7.1%), and blogs (*n* = 3; 5.4%). This was further supported with respondents reporting low use by lecturers of ICTs such as mobile phones (*n* = 31; 55.4%) (Table 3).

**Institutional support:** Respondents were asked about the ICT support they were receiving for using mobile phones and applications, such as Facebook at work and at institutions for educational purposes. Support for the use of mobile phones and social networking sites at university and work places was generally reported as poor, ranging from 9 (16.1%) for social networking to 34 (60.7%) for mobile phone support (Table 3). When examining whether mobile phones or social networking sites were used for educational and work-related purposes, more than half of the respondents (*n* = 32; 57.1% and *n* = 34; 60.7% respectively) reported that those functionalities were not supported at work.

## Individual characteristics

There is evidence (Gagnon *et al.* 2012) that the following individual characteristics are good indicators of future use of technology: perceived levels of competency, perceived effectiveness of technology, and general attitudes towards the use of ICTs.

**Perceived competency:** The ICT device that the respondents were most competent with was the mobile phone (Table 4). This finding was supported by additional findings that only 23 (41.1%) of the respondents reported that their friends would describe them as interested in the latest technology, and only 30 (53.6%) reported that they found technology easy to use.

**TABLE 4:** Individual characteristics (*N* = 56).

Variable	Agree (%)
Perceived competency	
Competent in the use of a mobile phone	40 (71.4)
Competent in the use of a smartphone to access the Internet	36 (64.3)
Competent in the use of a desktop computer/laptop to access the Internet	34 (60.7)
Generally competent in the use of a desktop computer/laptop	26 (46.4)
**Perceived effectiveness of ICTs**	**-**
Laptop	43 (76.8)
LMS (e.g. Moodle)	41 (73.2)
E-learning	34 (60.7)
Course related websites	34 (60.7)
Smartphone	31 (55.4)
Video	26 (46.4)
Mobile phone	22 (39.3)
Desktop computer	20 (35.7)
Camera	17 (30.4)

**Perceived effectiveness of ICTs:** In contrast, when respondents were asked to rate the perceived effectiveness of various technologies for learning, most respondents agreed that the laptop was effective (*n* = 43; 76.8%) followed by an LMS (*n* = 41; 73.2%), whilst less respondents agreed that the smartphone (*n* = 31; 55.4%) and mobile phone (*n* = 22; 39.3%) were effective (Table 4).

**Attitudes:** Respondents’ attitudes towards the use of ICTs in education were measured with regard to their own learning, their attitudes towards the use of ICTs at the university, and their perceived use of ICTs by their facilitators. The respondents’ attitudes towards using ICTs for learning showed that most respondents felt technological devices could be very helpful to access information (*n* = 42; 75.0% agreed) but less than half (*n* = 25; 44.6%) still felt that using technological devices was more helpful than frustrating (Table 5). Regarding the use of technology by the university and the facilitators, the respondents felt that technology was essential for learning (*n* = 41; 73.2%) and that the university used technology appropriately (*n* = 36; 64.3%). However, more investment was needed (*n* = 33; 58.9%) and although facilitators used technology effectively (*n* = 45; 80.4%) and frequently (*n* = 43; 76.8%), they did not know how to use technology that was available (*n* = 28; 50.0%) (Table 5).

**TABLE 5:** Attitudes towards the use of technology and education (*N* = 56).

Variable	Agree (%)
**Beliefs about technology and learning**	
Information technology has changed the way midwives access information	44 (78.6)
Information technology assists me with looking up information independently	42 (75.0)
Information technology assists me with verifying information from lecturers	41 (73.2)
Technology is essential for successful learning	41 (73.2)
I consider the trustworthiness of information I find on the Internet	40 (71.4)
Technology is more helpful than frustrating	25 (44.6)
**Beliefs about technology at the institution**	
Technology makes facilitators better at their job	43 (76.8)
Technology is essential for successful teaching	42 (75.0)
The university uses the technology effectively	36 (64.3)
The university needs to ensure the availability of more technology at rural sites	29 (51.8)
Technology is worth the investment	33 (58.9)
The university needs more technology	33 (58.9)
**Beliefs about instructors and technology**	
My facilitators use technology effectively	45 (80.4)
My facilitators use technology frequently enough	43 (76.8)
My facilitators know how to use the technology that is available	28 (50.0)
**Beliefs about technology (mobile phones)**	
Importance of using a mobile phone to discuss educational topics with facilitators	46 (82.1)
Importance of using a mobile phone to discuss work-related topics with facilitators	46 (82.1)
Importance of mobile phones to search for work-related information	43 (76.8)
Importance of using a mobile phone to discuss work-related topics with students	41 (73.2)
Importance of using a mobile phone to discuss work-related topics with colleagues	39 (69.6)
Mobile phones have changed the way I learn	35 (62.5)
Mobile phones are important tools in my daily life	30 (53.6)
**Beliefs about technology (Facebook)**	
Social networking sites have changed the way I learn	35 (62.5)
Using Facebook for medical topics supports my learning	13 (23.2)

The respondents’ attitudes towards the possible future use of mobile phones and Facebook for educational purposes were measured (Table 4). Though only half the respondents viewed mobile phones as important in their daily lives, mobile phones were rated important with regard to discussing learning and accessing information (*n* = 44; 78.6%). Respondents were positive about the use of social networking sites to assist their education (*n* = 31; 55.4%), however the specific ratings for Facebook were poor; only 13 respondents (23.2%) reported that they were using Facebook for obtaining medical information (Table 5).

## Discussion

In the context of using mobile phones and social networks to provide access to educational learning opportunities in lowly resourced settings for nurses, the following factors were considered: the specific rural context, the learners, and the potential applications and perceptions of these technologies for learning purposes.

Owing to insufficient access to the Internet at homes in the rural areas, mobile phones provide an opportunity to increase access to the Internet for learners. In South Africa mobile phones offer access to the Internet more readily than fixed line Internet connections at home, specifically in rural households where 17.9% of rural households access the Internet by using mobile devices as opposed to home Internet connection (2%) (Statistics South Africa 2013). In our study, a similar pattern was confirmed with low personal computer ownership. Unfortunately, rural hospitals in KwaZulu-Natal had very limited Internet access with a very slow bandwidth of 148 kbps (Wooton *et al.* 2009). In contrast, all except two respondents in this study reported that they owned a mobile phone. Half of them specifically reported owning smartphones that were used to access the Internet.

A second issue concerns the characteristics of nursing learners resident in rural areas, specifically regarding age and attitude towards the use of new technology. Owing to the fact that the respondents in this study were already qualified professional nurses, they were older learners with an average age over 40 years with 16% of the respondents over the age of 50 years. This finding was similar to the findings of other studies conducted in nursing at these settings (Chipps *et al.* 2015). Older nurses (nurses with extensive experience) might be more reluctant to embrace new technologies (Putzer & Park 2010), and age is known to be a factor that significantly affects computer competency levels (Hsu *et al.* 2009). Our study supported these findings, with more than half the respondents who reported low levels of computer competency and difficulty in using new technology. Generally, however, respondents felt relatively competent in using their mobile phones, though less so with new technological tools such as Facebook, with less than half of them ever having used Facebook. Only one of the Facebook users was over 50 years old.

Attitudes towards using a smartphone and technology are reported to have influenced the intention to use technology such as a smartphone (Chen, Park & Putzer 2010; Gagnon *et al.* 2012; Putzer & Park 2010). Generally, the respondents had a positive attitude towards technology and investments in technology by universities and their work place, though their views in respect of the use of those technologies for educational purposes remained out of date. Most of them rated the laptop as the most effective ICT for education and learning, followed by learning management systems and smartphones.

In assessing the potential for using mobile phones and social networks for learning at the time of the study, respondents’ mobile phone usage patterns, specific views on the use of mobile phones and social networks for education purposes, and institutional support were examined. The use of mobile phones and smartphones is increasing in health care provision with 64% of American physicians in 2009 using smartphones (Gill, Kamath & Gill 2012) and more than three-quarters of nurses in a recent study acknowledging that they often use their personal mobile phone or other communication devices at work, excluding during breaks or meal times (McBride, LeVasseur & Li 2015). The respondents in our study reported higher use of mobile phones compared to any of the other ICTs for educational and work-related activities, including contacting facilitators or peers to discuss topics and tasks related to the programme or work and searching for information. Making phone calls was the most frequent activity followed by SMS or texting, but they reported low current usage of smartphone functions.

With reference to social networks, the low engagement on Facebook in our study contradicts international studies of younger nurses with reports in the USA of up to 90% engagement on Facebook (Ferguson 2013). This is viewed as being related to the age of the nurses. Although, similar to international studies (Ferguson 2013; Schmitt, Sims-Giddens & Booth 2012) the respondents reported that social networking sites such as Facebook were becoming important in the nursing profession and education, regular use in our study to reflect on work-related experiences was low. Mobile phones were rated as important with regard to discussing learning and accessing information, and respondents were positive about the use of social networking sites to augment their education. However, the specific ratings for Facebook were poor. This could possibly be due to a perception that mobile phones and Facebook are not used for educational and clinical purposes and concerns about the potential distraction of those devices in a working environment (McBride *et al.* 2015).

Numerous studies have found that adoption of new technologies is largely dependent on the internal support of organisations (Bhattacherjee & Hikmet 2008; Putzer & Park 2010). The positive views towards potential use of mobile phones for learning were in direct opposition to reported poor support for the use of social networks or mobile phones at university or work. This was further compounded by a perception of lecturers’ poor awareness of the latest technology available for education and learning.

### Limitations

The study had a number of limitations. The main limitation was the 50% response rate which might have influenced the findings as a result of the non-responders’ own disposition towards using ICTs. Likewise, there was uncertainty about whether the respondents clearly understood the dissimilarity between mobile phones with smartphone capacities and traditional mobile phones.

### Recommendations

A number of recommendations emerged from this study. Firstly, there is empirical evidence from studies that the potential exists to use mobile phones and social networks more systematically in lowly resourced settings to facilitate nursing education. Institutions should evaluate how mobile phones and social media can contribute to key aspects of education, how to integrate them, and how to support learners to use them effectively. Specifically, orientation and training of older learners should be done to raise their awareness and skills in using mobile and social networks. Nursing education should invest in developing guidelines for the use of mobile phones in the clinical settings (Royal College of Nursing 2012). However, more formal research into possible strategies and interventions to facilitate the uptake of technologies in education in these setting is recommended.

## Conclusion

The study revealed an inherent tension and struggle between respondents’ relatively high usage patterns of mobile phones and high expectations with regard to their value for learning and work purposes on the one hand, and their limited perceived technical competency and the restricted institutional support on the other. Thus, it is concluded that much would be gained when educational institutions start supporting the learners with the use of mobile phones more systematically, particularly concerning the development of mobile literacy skills.
